# Sesquiterpenoids from the Herb of *Leonurus japonicus*

**DOI:** 10.3390/molecules18055051

**Published:** 2013-04-29

**Authors:** Liang Xiong, Qin-Mei Zhou, Cheng Peng, Xiao-Fang Xie, Li Guo, Xiao-Hong Li, Juan Liu, Zhao-Hua Liu, Ou Dai

**Affiliations:** 1 State Key Laboratory Breeding Base of Systematic Research, Development and Utilization of Chinese Medicine Resources, Sichuan Province and Ministry of Science and Technology, Chengdu 610075, Sichuan, China; E-Mails: xiling0505@126.com (L.X.); xxf14544@163.com (X.-F.X.); gli64@sina.com (L.G.); 2 Pharmacy College, Chengdu University of Traditional Chinese Medicine, Chengdu 610075, Sichuan, China; E-Mails: zhqmyx@sina.com (Q.-M.Z.); lixiaohong136@126.com (X.-H.L.); liuj755088@163.com (J.L.); 3 Chengdu No.1 Pharmaceuticlco. Ltd., Chengdu 610031, Sichuan, China; E-Mail: liuzhaohua01@126.com

**Keywords:** *Leonurus japonicus*, sesquiterpenoids, bioactivities

## Abstract

Two new sesquiterpenoids, (−)-(1S*,2S*,3R*)-3-ethoxycupar-5-ene-1,2-diol (**1**) and (−)-(1S*,4S*,9S*)-1,9-epoxybisabola-2,10-diene-4-ol (**2**), along with six known compounds **3**−**8**, were isolated from the EtOH extract of the herb of *Leonurus japonicus*. Their structures were elucidated by physical and spectroscopic analysis. In the *in vitro* assays, compounds **7** and **8** showed obvious antibacterial activity against several bacteria strains, while compound **3** significantly inhibited abnormal increase of platelet aggregation induced by ADP.

## 1. Introduction

Species of the genus *Leonurus* (Labiatae) are widely distributed in Eurasia, from Western Europe to China [1]. A number of bioactive secondary metabolites, including alkaloids [2,3], phenylethanoid glycosides [1], iridoid glucosides [4], cyclic peptides [5], diterpenoids [6,7,8,9] and triterpenoids [10], have been reported from several plants of this genus. *Leonurus japonicus* (synonyms *Leonurus heterophyllus*) is commonly used in Chinese herbal medicine for regulating menstrual disturbance, invigorating blood circulation, diuretics, and dispel edema [11,12]. In our previous study, chemical composition and antibacterial activity of essential oils from different parts of *L. japonicus* have been investigated [13]. The result showed that the oil of the herb (“Yimucao” in Chinese) had antibacterial activity against various Gram-positive bacteria and mainly consisted of sesquiterpenes and diterpenes, while the oil of the fruit (“Chongweizi” in Chinese) mainly made up of bornyl acetate and aliphatic hydrocarbons was inactive in the antibacterial assay. In searching for bioactive natural products from *L. japonicus*, we carried out a continuing investigation of the ethanolic extract of “Yimucao”. Two new sesquiterpenoids **1**−**2** and six known compounds were isolated from the EtOAc soluble portion of the ethanolic extract. This paper describes the isolation, structure elucidation, and bioassays of these isolates.

## 2. Results and Discussion

**Figure 1:**
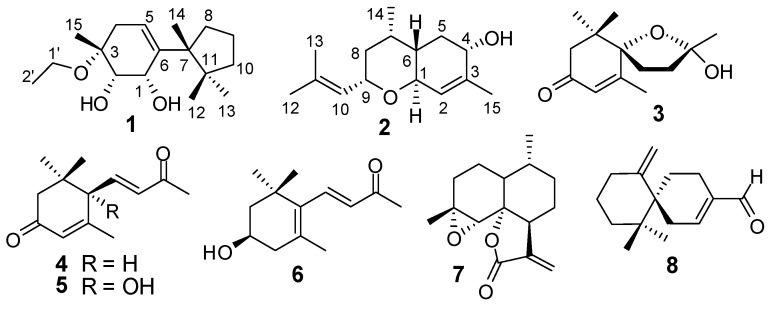
Structures of compounds **1**–**8**.

The EtOH extract of the herb of *L. japonicus* was suspended in water and successively partitioned with EtOAc and *n*-BuOH. Separation of the EtOAc fraction by column chromatography provided compounds **1**−**8** (Figure 1). The known compounds **3**−**8** were identified by comparing the spectroscopic data with those reported in the corresponding literature as (2*S*,5*S*)-2-hydroxy-2,6,10,10-tetramethyl-1-oxaspiro[4.5]dec-6-en-8-one (**3**) [14], 3-oxo-*α*-ionone (**4**) [15], (+)-dehydrovomifoliol (**5**) [16], (+)-3-hydroxy-*β*-ionone (**6**) [17], arteannuin B (**7**) [18], chamigrenal (**8**) [19].

**Figure 2:**
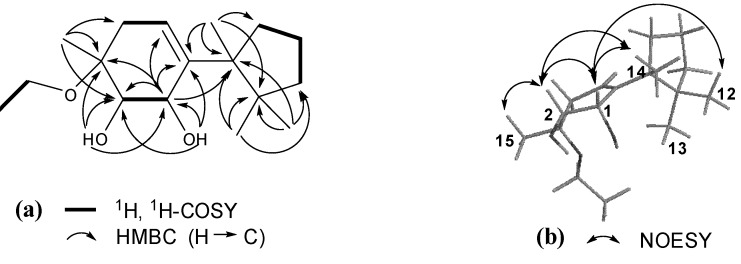
(**a**) Key ^1^H, ^1^H-COSY and HMBC correlations of **1**; (**b**) Key NOESY correlations of **1**.

Compound **1** showed IR absorptions for hydroxyl (3,418 cm^−1^) and olefinic (3,039 and 1,463 cm^−1^) functionalities. The molecular formula C_17_H_30_O_3_ of **1**, with three hydrogen deficiencies, was indicated by HR-ESI-MS and NMR data. The ^1^H-NMR spectrum of **1** displayed resonances attributable to four tertiary methyl groups [*δ*_H_ 0.82 (H_3_-12), 1.00 (H_3_-13), 1.06 (H_3_-14), and 1.25 (H_3_-15)], an ethoxyl group [*δ*_H_ 1.07 (t, *J* = 6.8 Hz, H_3_-2′), 3.49 (m, H-1′a), and 3.41 (m, H-1′b)], two oxymethines [*δ*_H_ 3.65 (m, H-2), 4.33 (m, H-1)], and an olefinic methine group [*δ*_H_ 5.62 (dd, *J* = 4.0 and 3.2 Hz, H-5)]. In addition, it showed resonances assignable to two exchangeable hydroxyl protons [*δ*_H_ 3.25 (d, *J* = 4.8 Hz, OH-1), 3.88 (d, *J* = 4.0 Hz, OH-2)] and partially overlapped resonances ascribable to several aliphatic methylenes between *δ*_H_ 1.40 and 2.40 (Table 1). The ^13^C-NMR and DEPT spectra of **1** revealed 17 carbon resonances (Table 1) corresponding to the above protonated units and four quaternary carbons (*δ*_C_ 45.9, 50.8, 76.1, and 143.1). These data suggested that **1** was a cuparene analogue with substitution of two hydroxyl groups and an ethoxyl group [20]. This conjecture was further confirmed by 2D NMR data analysis. The gHSQC spectrum of **1** furnished assignments of the proton-bearing carbon and corresponding proton resonances in the NMR spectra (Table 1). In the ^1^H-^1^H gCOSY spectrum of **1**, homonuclear coupling correlations of H-1/H-2, H_2_-4/H-5, and H_2_-8/H_2_-9/H_2_-10 revealed the presence of structural units containing the vicinal coupling protons (Figure 2). In the HMBC spectrum, correlations of H-1/C-2, C-3, C-5, and C-6; OH-1/C-1, C-2, and C-6; H-2/C-3, C-4, C-6, and C-15; OH-2/C-1, C-2, and C-3; H-5/C-1, C-3, C-4, C-6, and C-7; H_3_-12 and H_3_-13/C-7, C-10, and C-11; H_3_-14/C-6, C-7, C-8, and C-11; H_3_-15/C-2, C-3, and C-4; H_2_-1′/C-3 (Figure 2), in combination with the shifts of these proton and carbon resonances, demonstrated a gross structure of 3-ethoxycupar-5-ene-1,2-diol for **1**.

**Table 1:** NMR data (*Ā*) for compounds **1** and **2** in acetone-*d_6_* ^a^.

No.	1		2	
	*δ*_H_	*δ*_C_	*δ*_H_	*δ*_C_
1	4.33 m	70.1	4.25 br d (9.6)	69.1
2	3.65 m	74.9	5.38 br s	127.1
3	–	76.1	–	139.3
4	2.30 dd (17.2, 4.0), 2.19 dd (17.2, 3.2)	35.8	3.93 br d (4.8)	66.8
5	5.62 dd (4.0, 3.2)	123.1	2.16 dd (15.0, 3.6), 1.70 ddd (15.0, 4.8, 3.6)	32.7
6	–	143.1	1.38 m	39.5
7	–	50.8	2.24 m	28.5
8	2.36 dd (8.4, 4.4), 1.71 dd (8.4, 3.2)	36.9	1.42 ddd (12.6, 3.6, 3.0), 0.92 dd (12.6, 1.2)	41.3
9	1.62 m	19.7	4.22 m	73.9
10	1.65 (overlapped), 1.44 dd (12.4, 4.0)	40.4	5.11 d (7.8)	128.4
11	–	45.9	–	133.8
12	0.82 s	26.7	1.65 s	18.4
13	1.00 s	23.9	1.67 s	25.7
14	1.06 s	25.1	0.95 d (6.6)	20.3
15	1.25 s	19.7	1.79 s	20.7
1′	3.49 m, 3.41 m	56.8		
2′	1.07 t (6.8)	16.5		
OH-1	3.25 d (4.8)	–		
OH-2	3.88 d (4.4)	–		

*^a^* ^1^H-NMR data were measured at 400 MHz for **1** and at 600 MHz for **2**, respectively. Proton coupling constants (*J*) in Hz are given in parentheses. ^13^C NMR data were measured at 150 MHz for **1** and **2**. The assignments were based on ^1^H-^1^H COSY, HSQC, and HMBC experiments.

The configuration of **1** was elucidated by the NOESY data analysis [20]. In the NOESY spectrum of **1**, correlations of H-1 with H-2, H_3_-12, and H_3_-14; and H-2 with H-1, H_3_-14, and H_3_-15 (Figure 2) indicated that the orientations of H-1, H-2, and H_3_-15 were consistent with those of H_3_-12 and H_3_-14, but opposite that of H_3_-13. Thus, compound **1** was determined as (−)-(1S*,2S*,3R*)-3-ethoxycupar-5-ene-1,2-diol.

**Figure 3:**
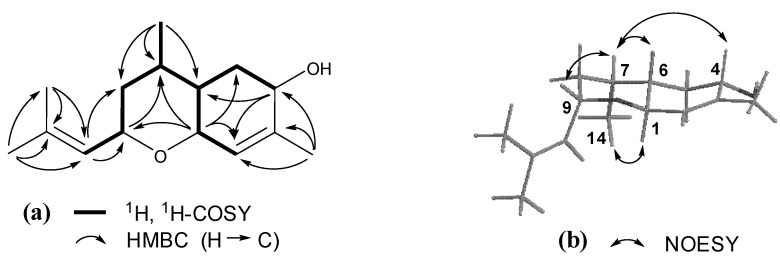
(**a**) Key ^1^H, ^1^H-COSY and HMBC correlations of **2**; (**b**) Key NOE correlations of **2**.

Compound **2**, obtained as a colorless oil, had the molecular formula C_15_H_24_O_2_ with four degrees of unsaturation as indicated by HR-ESI-MS *m*/*z* 259.1668 [M+Na]^+^ (calcd for C_15_H_24_O_2_Na, 259.1674). The ^1^H-NMR spectrum of **2** (Table 1) showed signals ascribed to a secondary methyl [*δ*_H_ 0.95 (d, *J* = 6.6 Hz, H_3_-14)] and three olefinic tertiary methyl [*δ*_H_ 1.79 (H_3_-15), 1.67 (H_3_-13), and 1.65 (H_3_-12)] groups, three oxymethines [*δ*_H_ 4.25 (brd, *J* = 9.6 Hz, H-1), 4.22 (m, H-9), and 3.93 (brd, *J* = 4.8 Hz, H-4)], and two trisubstituted double bonds [*δ*_H_ 5.38 (brs, H-2) and 5.11 (d, *J* = 7.8 Hz, H-10)]. In addition, the proton signals attributed to aliphatic methylenes and methines between *δ*_H_ 0.90 and 2.30, together with the ^13^C-NMR and DEPT data, indicated the presence of two aliphatic methylenes and two methines in **2**. These spectroscopic features suggested that **2** was a sesquiterpene and similar to (+)-bisabola-2,10-diene[1,9]oxide [21]. Comparison of their NMR data showed replacement of one methylene unit in (+)-bisabola-2,10-diene[1,9]oxide by an oxymethine (*δ*_H_ 3.93 and *δ*_C_ 66.8) in **2** (Table 1). Meanwhile, the olefinic proton signal for H-2 was changed from a doublet (*J*_1,2_ = 6.5 Hz) in (+)-bisabola-2,10-diene[1,9]oxide into a broad singlet in **2** (*J*_1,2_ ≈ 0 Hz). All the above spectroscopic data analysis indicated that **2** was an analogue of (+)-bisabola-2,10-diene[1,9]oxide with an additional hydroxy at C-4 and different configuration at C-1, which was proved by the 2D NMR experiments that amended the assignments of the NMR data (Figure 3). In the NOE difference spectrum of **2**, irradiation of H-7 enhanced H-4, H-6, and H-9, while H-1 was enhanced upon irradiation of H_3_-14 (Figure 3). These enhancements revealed that the protons H-4/H-6/H-7/H-9 had to be on the same side of the ring system, H-1/OH-4/isobutenyl-9/H_3_-14 were located on the opposite side. Therefore, compound **2** was determined as (−)-(1S*,4S*,9S*)-1,9-epoxybisabola-2,10-diene-4-ol.

The antibacterial activity of the isolates was assayed by the micro-dilution method [22]. Compound **7** showed the obvious activity against *Escherichia coli* and *Enterobacter aerogenes* with the MIC values of 25 μg/mL and 50 μg/mL, respectively, while compound **8** had the antibacterial activity against three Gram-positive strains, including *Macrococcus caseolyticus*, *Staphylococcus auricularis*,and *Staphylococcus aureus* (MIC 25, 50, 200 μg/mL, respectively). In addition, the inhibitory activity of the compounds against platelet aggregation induced by ADP was conducted by Born’s method [23]. The maximum aggregation ratio of the blank control was 61.4 ± 9.44%, while compound **3** evidently inhibited abnormal increase of platelet aggregation at a concentration of 10 μM, with the maximum aggregation ratio of 42.0 ± 15.63% (*p* < 0.01).

## 3. Experimental

### 3.1. General

NMR spectra were recorded on a Bruker-AV-400 or SYS-600 spectrometers. HRESIMS were measured with Waters Synapt G_2_ HDMS. IR were recorded on a Vector 22 FT-IR spectrometer. UV spectra were obtained on a Shimadzu UV-260 spectrophotometer. Optical rotations were measured with a Perkin-Elmer 341 plus. Platelet aggregation was recorded on Labor APACT-2 aggregation meter. Column chromatography was performed with silica gel (200–300 mesh, Yantai Institute of Chemical Technology, Yantai, China), MCI gel CHP 20P (75–150 μm, Mitsubishi Chemical, Co., Tokyo, Japan), and Sephadex LH-20 (Amersham Pharmacia Biotech AB, Uppsala, Sweden). HPLC separation was performed on an instrument consisting of a Cometro 6000LDS pump and a Cometro 6000PVW UV/VIS detector with an Ultimate (250 ƀ 10 mm) preparative column packed with C_18_ (5 μm). TLC was carried out with glass precoated silica gel GF_254_ plates (Qingdao Marine Chemical Inc., Qingdao, China).

### 3.2. Plant Material

The herb of *L. japonicus* (“Yimucao”) was collected in May of 2012 from the field in Wenjiang District, Chengdu City, Sichuan Province, China. Plant identity was verified by Prof. Min Li (Chengdu University of TCM, Sichuan, China). A voucher specimen (SYMC-0522) was deposited at the School of Pharmacy, Chengdu University of TCM, China.

### 3.3. Extraction and Isolation

The air-dried herb of *L. japonicus* (20 kg) was extracted three times with 95% EtOH (3 × 160 L) at room temperature for 72 h. The ethanolic extract was evaporated under reduced pressure to yield a dark brown residue (1.2 kg). The residue was suspended in H_2_O and then successively partitioned into EtOAc (400 g) and *n*-BuOH (160 g) fractions. The EtOAc extract (400 g) was subjected to silica gel CC using a gradient elution of petroleum ether–acetone (100:1–0:1) to afford nineteen fractions (F_1_-F_19_). F_4_ was further separated by silica gel CC over petroleum ether–EtOAc (35:1) yield four subfractions (F_4-1_–F_4-4_). The successive separation of F_4-3_ with Sephadex LH-20 (petroleum ether-CHCl_3_-MeOH, 5:5:1) and with PTLC (petroleum ether-EtOAc 10:1) yielded **4** (14 mg) and **8** (120 mg). Eluting with a step gradient of 50%-100% MeOH in H_2_O, F_7_ was separated by flash chromatography over MCI gel, to give ten subfractions (F_7-1_-F_7-10_). F_7-2_ was puriﬁed via Sephadex LH-20 (petroleum ether-CHCl_3_-MeOH, 5:5:1) to give F_7-2-1_–F_7-2-4_. F_7-2-2_ was fractioned via PTLC (petroleum ether-acetone 8:1) followed by reversed-phase semipreparative HPLC (75% MeOH in H_2_O) purification to afford **3** (15 mg), **5** (4 mg), and **6** (3 mg). Separation of F_7-2-3_ by PTLC (petroleum ether-acetone 6:1) and reversed-phase semipreparative HPLC (60% MeOH in H_2_O) successively yielded **2** (2 mg) and **7** (20 mg). F_7-3_ was separated by silica gel CC over petroleum ether–acetone (20:1–1:1) to get F_7-3-1_–F_7-3-6_. F_7-3-1_ was further puriﬁed by reversed-phase semipreparative HPLC, using MeOH–H_2_O (85: 15) to afford **1** (11 mg).

(−)-(1S*,2S*,3R*)-3-ethoxycupar-5-ene-1,2-diol (**1**): Colorless oil, 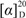 = −5.0 (*c* = 0.10, MeOH); IR (KBr) ν_max_: 3,418, 3,039, 2,963, 2,928, 2,874, 1,462, 1,368, 1,237, 1,096, 1,057 cm^−1^; ESI-MS *m/z* 305.2 [M+Na]^+^; HRESI-MS: *m/z* 305.2090 [M+Na]^+^ (calcd for C_17_H_30_O_3_Na, 305.2093); ^1^H- and ^13^C-NMR data see Table 1.

(−)-(1S*,4S*,9S*)-1,9-epoxybisabola-2,10-diene-4-ol (**2**): Colorless oil, 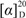 = −2.2 (*c* = 0.15, MeOH); IR (KBr) ν_max_: 3,478, 3,019, 2,925, 2,858, 1,461, 1,375, 1,230, 1,028 cm^−1^; ESI-MS *m/z* 259.2 [M+Na]^+^; HRESI-MS *m/z* 259.1668 [M+Na]^+^ (calcd for C_15_H_24_O_2_Na, 259.1674); ^1^H- and ^13^C-NMR data see Table 1.

### 3.4. Antibacterial Activity Experiments

All bacteria were obtained from clinical samples and stored in the Department of Pharmacology of Chengdu University of TCM. The *in vitro* antibacterial activity was determined by the standard agar dilution method, according to NCCLS (National Committee for Clinical Laboratory Standard) [22]. 5 μL of cultures of test strains at the concentration of 1 × 10^6^ CFU/mL were inoculated on Mueller Hinton agar containing different concentrations of the test compounds. The MIC values were determined after incubation at 35 °C for 24 h.

### 3.5. Platelet Aggregation Assay

SD rats were lightly anesthetized with ether. Blood was immediately taken from the femoral artery and anticoagulated with 3.8% trisodium citrate (9:1, v/v). Platelet rich plasma (PRP) was obtained by centrifugation of the whole blood at 800 *g* for 10 min. The precipitate of PRP was further centrifuged at 3000 *g* for 10 min to obtain platelet poor plasma (PPP). PRP was adjusted with PPP to about 2 × l0^8^ ~ 4 × 10^8^ platelets/L. Then, the platelet aggregation induced by ADP (final concentration: 0.05 mg/mL) was recorded on a dual sample aggregation meter according to Born’s method [23]. The antiplatelet efficacy was evaluated by comparing maximum aggregation response of the tested compound groups with that of control group.

## 4. Conclusions

Based on our previous study on the essential oil of *L. japonicus* obtained by hydrodistillation [13], we carried on a continuing examination of the EtOAc soluble portion of the ethanolic extract of the herb of this plant. Two new sesquiterpenoids, (−)-(1S*,2S*,3R*)-3-ethoxycupar-5-ene-1,2-diol (**1**) and (−)-(1S*,4S*,9S*)-1,9-epoxybisabola-2,10-diene-4-ol (**2**) were isolated, together with six known sesquiterpenoids. Among them, compounds **7** and **8** showed antibacterial activity against several bacteria strains, including *E. coli, E. aerogenes, M. caseolyticus, S. auricularis*, and *S. aureus*, with the MIC values in the range of 25 to 200 μg/mL. In addition, at a concentration of 10 μM, compound **3** displayed the inhibitory activity against platelet aggregation induced by ADP. According to the literature survey, cuparane- and chamigrane-type sesquiterpenoids were isolated from the genus *Leonurus* for the first time.
